# Playfulness and the meaningful life: an active inference perspective

**DOI:** 10.1093/nc/niad024

**Published:** 2023-11-18

**Authors:** Julian Kiverstein, Mark Miller

**Affiliations:** Department of Psychiatry, Amsterdam University Medical Research, Meibergdreef 9, Amsterdam South East 1105AZ, The Netherlands; Monash Centre for Consciousness and Contemplative Studies, Monash University, 29 Ancora Imparo Wy, Clayton VIC 3168, Melbourne, Australia; Psychology Department, University of Toronto, 100 St. George Street, 4th Floor, Sidney Smith Hall, Toronto, ON M5S 3G3, Canada

**Keywords:** playfulness, meaning in life, active inference, predictive processing, tolerance of uncertainty, mindfulness

## Abstract

Our paper takes as its starting point the recent proposal, at the core of this special issue, to use the active inference framework (AIF) to computationally model what it is for a person to live a meaningful life. In broad brushstrokes, the AIF takes experiences of human flourishing to be the result of predictions and uncertainty estimations along many dimensions at multiple levels of neurobiological organization. Our aim in this paper is to explain how AIF models predict that uncertainty can sometimes, under the right conditions, be conducive to the experiences of flourishing. Our focus is on playfulness, because playful individuals have learned a high-level prior that in certain safe contexts, uncertainty and error should be tolerated and explored. They have expanded the phenotypic bound on the amount of surprise they are prepared to tolerate in their lives. The positive embracing of uncertainty has a number of positive knock-on effects for the kind of lives playful individuals are able to lead. First, a playful individual attends to the world in a way that is open and expansive, a mode of attending that is effortless and therefore conducive to being in the present. This openness to the present moment allows for deep engagement and participation in experience that can furnish a renewed appreciation for life. Second, playful individuals will actively seek out spaces at the edge of their own abilities and will therefore be more likely to grow and develop in their skills and relationships in ways that contribute to their living a good life. Finally, playful agents seek out situations in which they can monitor, observe, and learn from their own affective responses to uncertainty. Thus, uncertainty becomes something familiar to them that they not only learn to tolerate but also enjoy positively exploring, in ways that provide them opportunities to grow. For these three reasons, we will argue that playfulness and openness to experiences of uncertainty and the unknown may be important ingredients in human flourishing.

## Introduction

Our paper takes as its starting point the recent proposal (Miller et al. 2022, Smith et al. 2022a), at the core of this special issue, to use the active inference framework (AIF) to computationally model what it is for a person to live a meaningful life. Such an explanatory project aims to address what Flanagan (2007) has called the ‘really hard problem’—the problem of explaining how it is possible for an evolved biological being, living in a material world, to live a meaningful life.

There are likely many factors that contribute to a person succeeding or failing to live such a life. Psychologists make an important conceptual distinction between eudaimonic and hedonic well-being (see e.g. Ryan and Deci 2001; Baumeister et al. 2013, Diener et al. 2017; Smith 2017, Bloom 2021). Eudaimonic well-being refers roughly to life-satisfaction—the capacity to fulfil and develop one’s potential through the pursuit of goals that fit with one’s personal values such as spending time with loved ones, working on one’s garden, making music, or campaigning for a political cause. Hedonic well-being by contrast focuses on momentary positive affect and the pleasures that arise from social interactions and physical activities like eating a delicious meal or drinking a fine wine. In what follows, we will sometimes also make reference to these distinct, and sometimes conflicting, components of flourishing.

By a ‘meaningful life’, we mean a life of flourishing, or what is sometimes called a ‘good life’. Peak experiences, social connectedness, mastery, and purpose have been argued to be important ingredients in flourishing (Seligman 2011; Smith 2017; Bloom 2021). We will however avoid committing to any particular account of flourishing. We agree with Bloom (2021) that there are likely to be many factors that contribute to flourishing and a great deal of individual variability. Our project is instead to explain what one can learn about human flourishing, broadly construed, using the computational modelling tools of the AIF. The AIF has as its theoretical underpinnings, the free energy principle (see Friston 2010, Parr et al. 2022). We do not discuss the free energy principle in what follows, though it is in the background of our discussion. We use the terminology of active inference, as a general unifying framework that applies to cycles of perception and action, and all the cognitive processes that depend on these cycles.

Our claim is that computational models of learning and action control can contribute to understanding some of the neurobiological processes that differences in experiences of human flourishing may depend upon (see Miller et al. 2022, Smith et al. 2022a). We will therefore assume that human flourishing is, at least partly, dependent on scientifically intelligible neurobiological processes that can be computationally modelled using the mathematics of the active inference theory. The active inference theory can be used to computationally model, and thereby to identify, the neurobiological causes and constituents that must be in place for a person to experience a life of meaning. Our aims in this paper are mainly theoretical and conceptual, to use AIF to identify a key ingredient—playfulness—that AIF would suggest might be a necessary ingredient for living a meaningful life.

In broad brushstrokes, the AIF takes experiences of human flourishing to be the result of predictions and uncertainty estimations along many dimensions, at multiple levels of neurobiological organization. AIF proposes that there are a limited set of states that must be maintained (through processes akin to homeostasis) if the creature is to persist in its current form in the future. This set of states are modelled as ‘expected’ states—states the organism believes it will continuously revisit. In order for these expectations to be fulfilled, the organism must act on its environment, seeking out for instance the warmth, food, security, and social attachment necessary for its continued existence. More generally, an individual’s desires, goals, and purposes are modelled as expectations. Expectations are beliefs or presuppositions about what events in the body and world are probable given, among other things, the individual’s goals, purposes, and desires. These beliefs serve a self-regulatory function, controlling the sampling of sensory information. They do so with the aim of maximizing the evidence for the agent’s beliefs, including beliefs about what is desirable, valuable, and worth pursuing in life. Since expectations take the form of beliefs, an agent that succeeds in maximizing the evidence for its beliefs will also succeed in fulfilling its expectations over time.

‘Uncertainty’ is translated in AIF terms as unpredictability relative to what is known. One might naturally think that unpredictability should be of negative value for creatures that aim at keeping their prediction errors to a minimum over time. This is indeed true for a certain kind of uncertainty experienced in volatile environments. Volatility can be analysed as unexpected uncertainty (Bland and Schaefer 2012) in which the outcomes of actions are continuously changing over time in ways that are hard to predict. Sustained exposure to volatility is associated with chronic stress, which leads to anxiety and a reduced sense of control (Barrett et al. 2016; Stephan et al. 2016; Paulus et al. 2019). In a volatile environment, the constant updating of predictions is necessary in ways that make it difficult to get a good grip on expected uncertainty.

Our aim in this paper is to use AIF to explain how uncertainty can sometimes, under the right conditions, be conducive to experiences of flourishing. In other words, unpredictability need not always be the source of negative affect and suffering. When playing for instance, humans and other animals enjoy creating just the right amounts of uncertainty (Andersen et al. 2022). The individual engaged in playing actively creates errors that violate their expectations, which they subsequently find a way to resolve. Thus, there is a slope of error creation and reduction in play. In our earlier work (e.g. Andersen et al. 2022), we have been focused on using the AIF to understand why play experiences are fun and enjoyable. In this paper, we will make a more ambitious claim. We will suggest that play does not only contribute to momentary happiness. Playful individuals have learned a high-level prior that in certain safe contexts, uncertainty and error can be tolerated. They have expanded the phenotypic bound on the amount of surprise they are prepared to tolerate in their lives. We will suggest that this ability to positively embrace uncertainty, when the conditions are right, may be an important ingredient in a life of meaning.

First, the playful individual attends to the world in a way that is open and expansive; a mode of attending that is effortless and therefore conducive to being in the present (Bar 2022). We can contrast this open experience of the present moment with a mode of relating to the world that prioritizes past habits, routines, and stereotypical modes of thinking. Open experiences of the present stand in contrast with a distracted state in which one’s awareness is taken up with oneself and the thoughts that are wandering through one’s mind. A playful openness to the present moment allows for deep engagement and participation in experience, which is, arguably, one important ingredient in flourishing.

Second, playfulness will have the consequence that an individual is prepared, in the right contexts, to hang out in volatile environments. Such agents will actively seek out spaces at the edge of their own abilities. They will be on the lookout for enough error to keep them interested but not so much error that the activity becomes uncontrollable and unmanageable. Agents that remain poised at the edge of their own abilities will be more likely to grow and develop in their skills and relationships in ways that contribute to their living a good life.

Third, we will describe how playful agents seek out situations in which they can monitor, observe, and learn from their own affective reactions. We focus on the example of engagement with fictive and highly improbable scenarios. When a movie goer watches a horror film, for instance, they experience events that are at once terrifying and also far removed from those they are likely to encounter in their everyday lives. They can nevertheless gain valuable lessons from engaging with such media about what it is like to experience intense disgust, fear, and anxiety at the very edge of what they can tolerate. What seems to motivate people to actively seek out such experiences is morbid curiosity—an interest in seeking information about highly threatening situations that trigger deeply rooted anxieties (Clasen 2017, Scrivner 2021a). We will suggest that engaging with our own emotions playfully can be functionally useful because it allows the person to learn about their own responses to uncertainty. Their responses to uncertainty can, in this way, become familiar and expected, something that can be well predicted, making these responses less newsworthy or attention-grabbing. Playfulness can thus contribute to emotion regulation, equipping a person with skills for engaging with the world in ways that are psychologically flexible. For these three reasons, we will argue that playfulness and openness to experiences of uncertainty and the unknown may be important ingredients in a meaningful life.

## Error dynamics and flourishing

Consider an unfortunate individual who consistently fails to achieve their goals and satisfy their needs. Such an individual will likely experience a mixture of anxiety and depression in their dealings with the world. Depression for instance has been linked to consistent failure to successfully regulate the internal conditions of the body leading to what has been termed reduced ‘allostatic self-efficacy’ (Stephan et al. 2016; see also Barrett et al. 2016; Paulus et al. 2019). Related experiences of ‘learned helplessness’ (Maier and Seligman 1976) then arise in which the person comes to experience that nothing they do can make a difference to achieving their goals and needs. This can be made sense of, in the terms of AIF, as the consequence of the person embodying a model whose predictions consistently fail to guide actions in ways that reduce expected predicted errors. Instead, the person comes to expect prediction errors—they no longer expect to achieve the outcomes they desire, leading them to remain in unhealthy relationships, or working a dull job that brings them no joy. They are certain of their own uncertainty about how to act in the world, which produces feelings of lack of control or helplessness (see Clark et al. 2018, Kiverstein et al. 2020).

The AIF predicts that it is not only the minimization moment by moment of prediction errors that is important for flourishing but also the minimization of *expected* prediction errors—errors arising from predictions that concern the person’s existence in the future. Keeping expected prediction errors to a minimum over time will sometimes require prediction errors to remain high at some lower layers of the generative model so that the expected prediction errors can be minimized at higher levels. Smith and colleagues give the example of high expenditure of metabolic energy during busy periods in one’s work life (Smith et al. 2022a). This expenditure of energy may well lead to high-allostatic load in terms of the stress and fatigue the person experiences. However, they may also find a sense of purpose and meaning in work that allows for the person to act in ways that are consistent with higher-level self-beliefs about what a good life means for them. Thus, even though errors are sustained at high levels at some layers of the model, this is the price to be paid for reducing expected prediction errors arising from higher-level self-beliefs that concern what a meaningful life looks like for them in the long-run.

Smith and colleagues have usefully mapped out six components that allow for a specification of what they call the ‘computational phenotype’ that underwrite different strategies for living a meaningful life. A computational phenotype refers to the values of the parameters of an internal model that best describe an individual’s behaviour (Schwartenbeck and Friston 2016, Smith et al. 2022a, Section 4). We will refrain from presenting the details of their account but we highly recommend interested readers to consult their paper. In earlier work, we have argued that one factor in both short-term happiness and long-term eudaimonic life-satisfaction is progress in error reduction (Miller et al. 2022). Drawing on Van de Cruys (2017), we have argued that valence depends upon error dynamics—the change in the rate of error reduction (Kiverstein et al. 2019a). By change in the rate of error reduction, we mean to refer to error reduction through action that unfolded either better or worse than the agent predicted. In selecting action policies that are expected to reduce prediction error, an agent must assess the efficiency of each action policy and pick the policy they believe to be most efficient. Rate of error reduction, we have hypothesized, may provide agents with a measure of efficiency (Kiverstein et al. 2019a). Reducing error at a faster than expected rate is an efficiency gain, while a slower than expected reduction in error means that an action policy performed less efficiently than was expected.

Affective states with positive valence (henceforth ‘positive affective states’) are elicited when the agent performs better than expected at bringing about preferred outcomes. This is to say that they do better than expected at reducing precise or significant errors, since preferred outcomes will be weighed as highly precise predictions. Conversely, affective states with negative valence (‘negative affective states’) occur when an agent does worse than they expected at securing preferred outcomes. An agent will experience negative affect when they fail to reduce a significant, precise error at the rate they expected.

To make progress in error reduction, an agent must be willing to disrupt habits of thinking and acting in ways that may lead to temporary increases in error and uncertainty. An agent must find the right balance between performing epistemic actions that aim at uncertainty reduction and pragmatic, habit-based actions that aim at directly and immediately bringing about preferred outcomes. A model that always opts for epistemic actions may well be accurate and comprehensive but it will fail to guide behaviour towards preferred outcomes in a particular action context. The agent will be faced with an explosion of possibilities they will need to prune in order to determine how to act in a particular context. A model that always generates pragmatic actions will run the risk of becoming trapped in local optima.

To escape these two extremes, what seems to be required is a context-sensitive weighing of the precision of predictions. A creature that does a good job of keeping prediction errors to a minimum over time must know when the predictions of its model are unreliable and not to be trusted and when to ignore prediction errors and to rely on its predictions. AIF assigns this work to so-called ‘precision estimations’ that regulate how much weight to give to prediction errors relative to predictions. When the quality of a model’s predictions is estimated to be high, any prediction errors will be down-weighted. Recall how the agent’s needs, goals, purposes, and preferences are modelled as expected sensory states—sensory states that the agent expects, with a high degree of confidence (i.e. a high-precision weighting), to be able to bring about through its actions. The agent’s model can be said to be ‘optimistically biased’ (Sharot 2011): the expectations that the agent estimates to have a high precision tend to relate to states that the agent optimistically believes with high confidence it can bring about through its actions.

If an agent is to succeed in keeping its prediction errors to a minimum, it is necessary that this optimism bias be kept in check by reality. The expectations the agent has about the future states they can affect through their actions must fit with what can realistically be achieved in the particular context in which they are acting. It is therefore important that the agent strikes the right balance between acting confidently to bring about its preferences and updating its beliefs about what goals it can expect to realize in a given context. Without finding this balance, and sometimes using feedback from reality to update its optimistic expectations, the agent will run the risk of an inefficient expenditure of its resources. Van de Cruys et al. (2020) describe the biasing of the predictive model by the agent’s needs and preferences as a ‘controlled optimism’. Predicted states corresponding to an agent’s goals, needs, and preferences must exhibit some robustness in the face of challenges. At the same time, it is also necessary that the agent must be open to revising some of these predictions when the evidence points to the predicted outcomes being unattainable.

We have proposed elsewhere, that this balance could be achieved by using affective states, understood in terms of error dynamics, to weigh precision as the agent shifts between contexts (Kiverstein et al. 2019a; and see Hesp et al. 2021 for modelling work that is supportive of this hypothesis). An agent that uses error dynamics to weigh precision and update its precision expectations as it moves between contexts will stay well-tuned to opportunities to make progress in learning. This is to say that they will do a good job at acting in ways that are conducive to bringing about preferred outcomes, arguably a key ingredient in living a good life. They will also tend to spend a good deal of their lives in positively valenced affective states, since valence is, we argue, dependent on error dynamics. Agents that tend to do better than expected at error reduction because they succeed in continuously finding their way to opportunities to do better than expected at bringing about their preferred action outcomes will likely feel good about their lives.

Of course, in real life, people rarely fare so well. The environment, which includes other people pursuing their own preferences and interests, is frequently uncooperative. The person must take the good with the more frequently occurring bad. Our point is simply that it is a prediction of AIF that tolerating error and uncertainty, through exploring for opportunities to improve one’s skills and abilities, is an important ingredient in human flourishing. The opposite may also be true: low tolerance for error leads to less exploration, which in turn leads us to pull back and retreat from volatile situations. This can have the consequence that the person’s beliefs become rigid making them more brittle and less able to maintain a good grip on a dynamically changing world. People that are less tolerant of error will tend to exhibit a strong need for control. When they find themselves in situations in which this need is not fulfilled, they may experience intense anxiety. One response to anxiety is to fall back on habitual modes of behaviour. This can be seen in the ritualistic and compulsive behaviours of people diagnosed with obsessive compulsive disorder that are a means to the reduction and control of the anxiety arising from their obsessions (Kiverstein et al. 2019b). Long-term addiction can also often be viewed as a reasonable strategy for keeping an otherwise unruly and volatile environment under control (Miller et al. 2020; Lewis 2015).

We will argue in the next section that play activities are examples of behaviours in which an agent productively tolerates and explores error. When playing, agents use error dynamics to weigh precision with the result that they are guided to what we have called ‘just-right’, consumable surprises—errors that are neither too complex nor too easily predicted such that there is nothing for the agent to learn. We will suggest that the disposition of playfulness may be an important ingredient in human flourishing. Playful individuals are prepared to tolerate uncertainty that accompanies temporary increases in error. It is this tolerance or equanimity towards uncertainty which we will focus on in the remainder of our paper as a key ingredient in flourishing.

## The joy of play

Animals and humans engage in play in large part because it is enjoyable and fun to play. Play is intrinsically motivated—it is rewarding in itself, not only as a means to bring about an extrinsic reward. Ryan and Deci (2000) contrast intrinsic with extrinsic motivation based on activities that are performed for ‘the enjoyment of the activity itself, rather than its instrumental value’. Think for instance of a child’s motivation for doing their homework (Oudeyer and Kaplan 2007). They can be extrinsically motivated by the threat of punishment—the sanctions from their parents and teachers—which they will receive if they do not do their homework. However, they may also be intrinsically motivated by the pleasure of learning, discovering new knowledge, or finding the solution to a puzzle. Solving a problem in mathematics can for instance be motivating for some students of mathematics in a similar way to playing a video game. Both can be intrinsically fun activities.

What is it that learning and play share in common such that both can be enjoyable activities? What makes an activity intrinsically fun? First, both learning and play can be characterized by active exploration or information-seeking that results in the reduction of uncertainty or better than expected gains in error reduction. Novel experiences (i.e. an unpredicted event that violates pre-existing expectations) can be intrinsically motivating if the sampling of observation leads to new discoveries that improve the future predictions of a model (Oudeyer and Kaplan 2007, Oudeyer et al. 2007). Agents that aim at prediction error minimization will be intrinsically driven to explore in ways that lead to temporary increases in error. In doing so, they will be led to new discoveries that allow them to do better than expected at reducing error in the long-run. Recall that doing better than expected at reducing significant (i.e. precise) error is, we have argued, the source of positive affect. Novel experiences that allow for learning progress will be intrinsically motivating because they elicit states with positive valence.

The promise of securing positive affective states will therefore drive agents towards niches replete with ‘consumable errors’—errors that are neither too complex to get a practical grip on nor too simple and predictable to be devoid of new information. In earlier work, we have argued that in play activities, agents willingly create errors and surprises that they can resolve over the course of the play activity and thereby make progress in their learning (Andersen et al. 2022, Deterding et al. 2022, Miller et al. 2023). When playing, people intentionally create situations in which they generate small consumable errors. They deliberately create situations in which they are uncertain what will happen, temporarily generating a sensory flow that takes them outside of their comfort zone. In an enjoyable game, there will not be so much unpredictability that the agent loses grip on the game. Nor should the game be so predictable that there is nothing of surprise that happens. The game should instead strike the sweet spot between predictability and unpredictability we have described in terms of the game or play activity offering ‘just-right’ surprises (Andersen et al. 2022). The surprises the agent creates pique their interest because they contain just the right mixture of novelty or violation of expectation.

Play allows agents to encounter new information with just the right amount of complexity for them to make progress in learning. This feature of games is interestingly related to what the developmental psychologist Lev Vygotsky (1978) called the ‘zone of proximal development’. Vygotsky showed that the developmental growth takes place at the edge of an agent’s current abilities. In the terms of the AIF, what we are calling ‘consumable errors’ allow for the learning of something new and thus for growth and improvement in one’s skills and abilities (i.e. for learning progress). A child for instance playing with a faucet will encounter surprise when they are sprayed with water. However, it is just the right amount of surprise for them to learn how to do better at reducing error in the future. Play feels good when the agent does better than expected at actively transforming an unpredictable and uncertain world into a predictable one. Video games, for instance, are often designed in such a way as to amplify, apportion, and sequence uncertainty so that the player can eventually, with some effort, reliably resolve their uncertainty, preferably at a faster than expected rate (Deterding et al. 2022). Such a carefully engineered game will build in possible jolts of positive affect as the player gradually experiences mastery of the game.

So far, we have discussed the contribution of play to momentary happiness. It might therefore be thought that play is only relevant to explaining meaning in life if one understands flourishing in hedonistic terms of maximizing pleasure. In the remainder of our paper, we will argue however that such an impression would be mistaken. Play might also contribute to long-term life-satisfaction. Here, what seems to be important is the more global trait of playfulness. This trait can be thought of as a disposition, a persistent background state of mind, that shapes how a person finds themselves in the world from moment to moment. Playful people tend to be curious; they are more inclined to investigate the world and learn about it. By ‘curiosity’, we mean the tendency to seek out novel and challenging interactions with the world in ways that facilitate learning and growth in skills and relationships (Kashdan and Steger 2007, Berlyne 1960). Playful individuals will expose themselves to new experiences in ways that temporarily increase their uncertainty but that also allow for growth in skills and relationships that we have been arguing are key ingredients in flourishing.

## Openness to experience

Playful people who are highly curious about the world, and about other people, will be more likely to be tuned into consumable errors. They will tend to engage in exploratory behaviours of the novel and the challenging, thereby making it more likely that they will encounter events that offer possibilities for growth and meaning in life (Kashdan and Steger 2007). What is crucial here is that the error and uncertainty the agent encounters be “consumable”—that is to say, at just the right level to be mastered given the individual’s competence. Consumable errors provide the agent with the opportunity to act at the edge of their current ability, mastering and resolving the uncertainties that arise and thereby growing in their skills. Playful individuals will tend both to be drawn towards errors and believe they are competent to get a good grip upon those errors. This combination of skills means that playful individuals will be well-positioned to take advantage of and make the most out of opportunities for growth when they arise.

Sadly adults, unlike children, tend not to have the trait of playfulness, shunning opportunities to explore uncertainty. Adults for instance will often approach going to the gym or doing sports as something to do because of its functional benefits, not as an opportunity to explore and grow in their abilities (Thiel et al. 2016). Why is this? We can provide a tentative answer to this question by contrasting children that tend to exhibit playfulness with adults who do not. Developmental psychologists hypothesize that one reason why children are motivated to play is that play gives them an active way to learn how the world works and what it affords for them. Children engage in what is called a ‘high-temperature’ search over a broad space of hypotheses that allows them to grow in their knowledge of the world and its workings (Gopnik 2020). Adults, by contrast, have thoroughly explored and experimented with the world earlier in their lives and only expend the effort to experiment and explore when they expect to learn something new. They tend to engage in low-temperature search over a narrow space of hypotheses, assigning high precision to their prior beliefs. The knowledge adults have amassed about the world over the course of their development can lead to our being somewhat inflexible and narrow-minded in our thinking. The more knowledge we amass, the more certain we become of how the world works. We tend to act based on a desire to exploit what is already known to plan and to get things done. The result is a mode of cognition characterized by ‘attentional focus, inhibition, and executive function and behaviours like long-term, goal-directed planned action’ (Gopnik 2020: p.2). Moreover, many of us grow up in cultures in which play is regarded as frivolous, unproductive, silly and even irresponsible when there are more serious matters to attend to. We learn from our peers that society expects us to be sensible, serious, and above all else productive, which leaves us with no room in our lives for enjoying playing. In the West, we also tend to structure our educational practices around such an expectation. There is increasingly little room in the classroom for play and active exploration due to a greater emphasis on standardized testing that leaves less room for thinking creatively and imaginatively (Kim 2011).

There is however a high price to pay. First of all, and perhaps most obviously, we miss out on the moments of joy and pleasure that can occur when we play. Worse still, we may run the risk of becoming rigid, narrow, and psychologically inflexible in our beliefs about the world if we do not remain open to exploring the world. The exploitative mode of cognition in which high precision is assigned to prior beliefs leads us to act based on habit and expectation. This allows us to keep our attention focused on narrow tasks which of course can be important for achieving our goals. However, inflexible precision weighting can limit the possibilities for a person. It can keep them locked into patterns of thinking and acting based on past experience that may not serve us well and that may also stand in the way of them changing. Just exploiting what has been learned in the past does not leave space for the kind of broad, open, and creative thinking that can result in progress and growth in skills.

The opposite of psychological inflexibility is open, curious, and broad exploration that allows us to have the kinds of novel experiences less tightly constrained and bound by past expectations. An example of this flexible psychological mode of thinking and acting is the state of mind that can be induced through the use of psychedelic drugs (see Carhart-Harris and Friston 2019). The relaxing of the precision on prior habits and expectations that occur with the use of psychedelic substances results in a flattening of the attractor landscape the person forms in their dynamic coupling with their environment (also see Hipolito et al. 2023, this volume). No attractor in this landscape is too dominant allowing for an agent to escape local optima and to engage in a wider exploration of possibilities. In particular, psychedelics relax the precision given to high-level beliefs the person may have formed about themselves and their past experiences in ways that make possible transformative experiences that change the person’s sense of who they are and what matters in their life.

Psychedelics introduce disorder into the brain (and the larger agent-environment system the person forms with the world) in such a way as to remould the rigid beliefs the person may have formed in the past about themselves and the world. They induce a state in which rigid and inflexible habits of thinking and acting become objects apt for reflecting on, testing, challenging, and reshaping. Something similar may also be true of the global background state of mind of playful individuals. They are constantly ready to stress, challenge, break, and rebuild the models that shape their perception of the world (Miller et al. 2020). Curious individuals can find it interesting to engage in behaviours that are incongruent with their habitual behaviour tendencies. They can experience positive affective states when engaging with novel and challenging situations that diverge from those that they have come to expect. Shedding the defences that one has set up over the course of one’s life to protect oneself can be enormously stressful. The playful person however is able to find their way to meaningful opportunities for growth precisely by being willing to shed these defences in ways that allow them to move forward and grow.

What is crucial is that the novel and challenging events they seek out are ones that they believe they are competent to manage and control. This self-confidence makes them more prepared to tolerate uncertainty and the resulting stress that might be experienced as aversive and to be avoided by the less playful among us. Indeed for the playful person, anxiety and fear are just more experiences to be curious about that can be rendered ‘familiar, predictable, manageable, and controllable’ (Maslow 1962, p.6, quoted by Kaufman 2020, p.93) through exploration that leads to growth in knowledge and understanding of self and world. The playful person’s high-tolerance of stress makes them more able to explore the unknown and uncertain, unimpeded by their fears and anxieties. This has the consequence that they are able to grow in their skills in ways we have been arguing contribute to them leading a meaningful life.

Kaufman (2023) has recently argued for a strong relationship between playfulness and self-actualisation, which he defines as the ‘motivation to become all that one is capable of becoming’. People scoring highly on playfulness are also likely to be motivated to pursue personal growth in their lives (See Kashdan et al. 2018 for a similar finding in a study investigating the relation between the curiosity trait and personal growth). Playful individuals that exhibit a need to explore the world for novel and challenging experiences have also been shown to exhibit reduced latent inhibition (Peterson and Carson 2000, Carson et al. 2003, Kaufman 2020: pp.110–11, cf. Bar 2022, pp.137–40). Latent inhibition refers to a preconscious gating mechanism associated with dopamine production in the brain (Lubow and Weiner 2010). It is thought to allow for the imposing of top-down prior conceptual categories onto the world in ways that constrain the activation of associations and the expansiveness of a person’s thinking. Latent inhibition serves the important function of allowing individuals to tune into what is relevant and what is not in a particular context, which is clearly crucial for acting in ways that call for a narrow focusing of attention, such as when driving under dangerous conditions. (Panksepp 1998) has suggested that rough and tumble play may promote frontal lobe maturation and areas of the brain associated with inhibition. Panksepp suggests that ‘[t]he explosion of ADHD diagnoses may largely reflect the fact that more and more of our children no longer have adequate spaces and opportunities to express this natural biological need to play with each other in vigorous rough-and-tumble ways, each and every day’ (p. 91)). Inhibition however also narrows our engagement with the world to what is relevant to meeting our immediate needs, goals, and preferences. Reduced inhibition leads to experiences that are not so tightly constrained by top-down expectations. Such experiences may allow for what Maslow (1954) described as a ‘continuous freshness of appreciation’. The deployment of attention is no longer guided by top-down predictions that are given high precision.

Intriguingly, this kind of quieting or turning down the precision on top-down prediction has been argued to be a feature of the type of conscious state people are able to enter into through engaging in practices of meditation and mindfulness (Laukkonen and Slagter 2021; Bar 2022). It allows people to be more present in the moment instead of experiencing the world exclusively through the lens of their past expectations and habits of thinking and acting. This may in turn allow them to experience the familiar afresh and with less distraction from their expectations that take them away from the moment unfolding in front of them.

Mindfulness meditation, in common with playfulness, enables psychological flexibility. By remaining in the present, the person need no longer get caught up with and stuck in unhealthy patterns of thinking and acting. Gaining some distance from their expectations means no longer identifying with them as fixed and unchanging properties of ourselves but as potentially changeable through reframing. In the final section of our paper, we describe how playful exploration of uncertainty can allow an agent to monitor and learn about their own affective responses. This learning can make opaque the affective processes that would typically transparently drive action policy selection. This process of opacification allows for the same kind of psychological distancing from our affective responses as have just described in mindfulness meditation. A playful person will be open to reframing and reinterpreting their habitual responses in ways that can allow them to grow in wisdom and intelligence.

## Play, metacognition, and wisdom

Much of the literature on active inference has tended to focus on two ways in which error can be reduced over time. Either the agent makes their model fit the world through updating the predictions of a model—the prior beliefs the model encodes—or they make the world fit the model by producing sequences of actions that bring about preferred (i.e. predicted) sensory outcomes. There is however a third, under-appreciated way in which to reduce the errors one encounters in the long-term (which was, incidentally, already highlighted by Clark (2013)). This is to change how precision is deployed. For example, faced with the uncertainty of a late train, we can update our model to form the belief that our train is late (perceptual inference), leaving unchanged our frustration. Alternatively, we can leave the station and take a taxi instead (active inference). Finally, we can view the lateness as an opportunity to have some more time to sit quietly on a bench at the train platform and enjoy a coffee (redeployment of precision). In the last possibility, we have found a way of reframing the situation, giving less weight to the frustration that would otherwise provide the framing for our perception of the late train. In fact, as we will see, it is by altering our experience of frustration (giving it less importance) that we improve our ability to change our perspective on the situation. Play (especially risky play) offers good opportunities to learn and change our relationship with uncertainty and the frustration it can bring.

We suggest that playful engagement with consumable errors (a safe amount of uncertainty) can help predictive agents to learn about how to redeploy precision in much the same way as in the train example. Consider as an example of such playful engagement with error, listening to true crime stories, or watching horror movies. People that enjoy these media are engaging with fictional realities depicting extremely low-probability but highly volatile and threatening events. What is interesting and makes us curious to explore such realities, despite the improbability of our ever finding ourselves in such situations, is our own embodied reactions. Play of this kind provides us with the opportunity to learn about our own reactions to dangerous and highly volatile situations in which our lives would be at risk were we to be so unfortunate to encounter them. Horror films provide people with the possibility to explore simulated or imaginary experiences of dangers and threats. What such fictions do is imagine what it would be like were one’s worst fears, and even fears one had never previously imagined, to be brought to life. This allows viewers to safely gather information about what it would be like to deal with dangerous situations, so as to prepare for related situations they may encounter in their own lives. Indeed, recent research has shown that people who watched horror movies during the pandemic experienced less psychological distress during the pandemic (Scrivner 2021b) and reported feeling more prepared for the second-wave (Gifford 2020).

There is evidence more generally that people who are attracted to horror movies tend also to experience high levels of anxiety. People diagnosed with anxiety disorders (such as generalised anxiety disorder, panic disorders, and social anxiety disorder) have been shown to also be fans of horror (Grisafi 2016; Turner 2017; Scrivner & Christensen). These individuals experience exaggerated and out of proportion worry and apprehension they find difficult to control in response to potentially threatening situations such as difficulties at work or in personal relationships. Morbid curiosity, in which people are motivated to seek out information about threatening situations, is no doubt part of what explains why people with high levels of anxiety in their lives might enjoy horror. Horror stories provide anxious people with information about the situations that worry them that may allow them to better prepare for these situations.

We suggest that in addition engagement with horror stories may provide highly anxious people with a means of affect regulation. Learning about our own affective reactions requires metacognitive monitoring—the observation of one’s own cognitive operations. Agents are learning to metacognitively model their own affective responses when they engage with fictive realities. We have suggested the valence of affective states can be identified with error dynamics that may play a role in the context-sensitive adjustment of precision weighting. When playful engaging with fictive realities allows a person to learn about their own affective responses, this amounts to the person learning to model their own deployment of error dynamics in precision weighting. They are learning second-order expectations about how precision is assigned across different contexts.

Competitive sports may also serve as a context in which to learn such second-order expectations about how precision is assigned. For example, in many competitive play experiences (e.g. football, baseball, martial arts, etc…) players are similarly learning about what it is like to act skillfully under pressure, which requires them to learn about, and so increasingly tolerate, a variety of challenging uncertainty-related experiences (e.g. the stress of performing well, supporting your team, dealing with forms of aggression, managing unexpected situations, avoiding/tolerating injury, etc…). Learning about how one responds to uncertainty can be important for flourishing, because it contributes to resilience and what Taleb (2012) calls ‘antifragility’—the growth of skills in the face of stress. (See Kiefer et al. (2018) for an example of these ideas in the context of training competitive athletes.) We suggest, in addition, that learning to tolerate stress and uncertainty can contribute to the learning of emotion regulation skills that are important for flourishing in life more generally. We will return to this point below.

Following Sandved-Smith et al. (2021), we suggest that as an agent increasingly models its own error dynamics it improves its ability to regulate those dynamics. The effect of learning about our affective responses is that we can potentially render prediction error signals less newsworthy that would otherwise be given high-precision weighting. We can turn down the precision on these error signals. This happens by making the error signals opaque. Ordinarily, error dynamics are experienced transparently—they are not accessible to the system itself, but rather drive selections of policies directly given our phenotype and habits. Insofar as negative affective states are transparent, they are treated as signalling sources of error that could be essential to the self’s integrity. They therefore get to drive the agent’s behaviour as if they were essential to the agent’s integrity. This has the effect of producing a powerful ‘urgency for change’ in the agent, leading it to behave in ways that either prolong the positive state or quickly bring to cessation the negative ones.

The opacification of this part of the precision machinery opens new opportunities for control. Observing the arc of our affective reactions in play allows us to develop new higher order policies about how precision (via valence) is being set on policies. Instead of affective states adjusting precision on policies directly, and so automatically conditioning us to behave in certain ways, one can now learn to activate alternative policies depending on the usefulness of the valenced signals. Consider the extraordinarily free-climbing skills of Alex Honnold. He has climbed some of the most challenging of America’s steep cliff-faces without ropes, harnesses, or protective equipment, including the 3000 foot El Capitan in Yosemite National Park. Honnold tells us he has trained himself to no longer be moved by fear. When he first began free-climbing, he experienced the very same fear responses signalling the danger of the sport that you or I would experience. Over time however he learned to have a high tolerance for uncertainty so that he is now able to continuously explore and stretch the limits of his own skills. We suggest this may be something Honnold was able to train himself to do by no longer relating to his negative affective states of fear and anxiety transparently as signals that should be given high-precision and therefore drive the selection of his actions. Instead, he made his negative affective states opaque, gaining sufficient psychological distance from them until they were no longer given the weight to automatically drive his behaviour. Interestingly, when Honnold was placed in an fMRI machine and presented with emotionally highly disturbing images, his brain showed virtually no activity in areas such as the amygdala that would typically be highly activated (Mackinnon 2016). It is as if in training himself to be fearless he succeeded in turning down the precision signals on error signals, expanding the phenotypic bounds on the amount of uncertainty, danger or threat that his body is prepared to tolerate.

Again a comparison with mindfulness will prove instructive. Mindfulness has been shown to be highly effective, in people who are trying to quit smoking cigarettes, at disrupting the habitual patterns of response that lead from the affective state of craving to smoking (Bowen and Marlatt 2009). Mindfulness in effect selects a policy to closely attend to the feelings of craving—the negative valence driving processing toward the expected rate of error reduction relative to nicotine levels—every time they arise. The new goal (instead of smoking a cigarette), now activated every time there is a craving, is to watch, as closely as possible, the arising, the progression, and the inevitable depletion of the craving-related feelings. Overtime, these sorts of mindfulness practices have the effect of making cravings opaque, teaching the system that cravings are in fact just feelings in the body, which can be allowed to direct processing and behaviour or not. This discovery can represent a major return of control for people struggling with nicotine addiction. The negative valence of cravings remains unchanged through this process of opacification. What changes is that it begins to be interpreted by the system as information that can be useful, but is not obligatory, in selecting policies.

We see then that as an agent learns to model their own processes of precision estimation, they also improve their ability to regulate their emotional reactions—the degree to which error dynamics are allowed to directly drive action selection. Sandved-Smith et al. (2021) have suggested that in a deep parametric generative model, lower-level precision dynamics (such as those that are believed to be closely related to affective valence) can be optimized by deeper predictions about precision deployment over predictions. In effect, the agent is now able to take lower-level precision estimates as the observations over which higher-level predictions can now be made. These meta-cognitive abilities increase our capacity to stay flexible—not driven directly by affective changes. This flexibility in turn allows us to more readily cognitive reframe like in the example of the late train with which we began. This ability to cognitively reframe requires there to be a tolerable amount of uncertainty about its expectations, which will allow the system to flexibly alter those expectations and so to change their behaviour.

This kind of psychological flexibility may be a key part of living wisely. Once an agent’s affective reactions become opaque, they will no longer unthinkingly drive the agent’s behaviour. The agent can gain sufficient distance from their affective reactions so that they no longer identify with their affective states as reflecting their current reality and how they are faring in the world. They can engage flexibly with their affective states without reifying or taking to be objectively real how those states present the world. Many psychopathologies, from addiction to depression, are characterized by an agent’s reifying their affective responses to the world. Think for instance of craving in addiction or of ruminative thinking in depression. Agents relate to their emotions transparently, taking them to represent reality rather than to be a particular interpretation or perspective on reality. As we saw in the previous section, the result is a rigidification of the agent’s priors laid down through past patterns of thinking and acting. The agent is no longer drawn into an open and broad mode of curious exploration. What is common to the dispositions of mindfulness and playfulness is an openness to explore the unknown that is a consequence of the person being prepared to tolerate uncertainty. The willingness to stress, break, and challenge their model of the world opens up the possibility to grow in their skills, which we have argued is a key element of living a meaningful life.

## Conclusion

We have used the AIF to identify a key computational ingredient in human flourishing—playfulness. We have argued that playfulness may contribute to living a meaningful life in three ways. In play activities, agents are drawn towards what we called consumable errors—errors that are complex and challenging but nevertheless manageable given the individual’s level of competence. We have argued first that agents that are disposed to engage with the world playfully will tend to seek out novel, challenging, and uncertain information. They will be willing to explore the unknown and unfamiliar without fear or anxiety. They will thereby find themselves well-positioned to take advantage of, and make the most out of opportunities for growth when they arise. Second, playfulness is characterized by a reduced latent inhibition. This means that the playful individual is able to encounter the familiar without expectation allowing for an experiencing of the world in the present moment and with fresh appreciation. Third, playful agents will tend to actively create situations in which they can monitor, observe, and learn from their own affective reactions. In learning to model their own affective reactions, agents can come to exercise improved emotion regulation. Their affective responses to the world become opaque, no longer unthinkingly driving action. Instead, the individual is able to flexibly reappraise their meaning. What is the connection between these three features of playfulness and flourishing? We suggest that what they share in common is that in each case the agent has learned to tolerate uncertainty. The playful individual has installed a high-level prior that uncertainty is sometimes safe to explore and even to create. The insight that uncertainty is neither necessarily good nor bad but is to be treated with an attitude of equanimity is, we have suggested, among the key ingredients in living a fulfilling and meaningful life.
